# Sport facility planning and development in the eyes of local residents

**DOI:** 10.3389/fspor.2026.1903765

**Published:** 2026-10-01

**Authors:** Szilvia Perényi, Balázs Polcsik

**Affiliations:** 1 Department of Sport Management, Hungarian University of Sports Science, Budapest, Hungary; 2 Institute of Physical Education and Sports Science, Juhász Gyula Faculty of Education, University of Szeged, Szeged, Hungary

**Keywords:** Budapest, forms of sport involvement, post-event use, residents’ perceptions, sport facility development, World Athletics Championships (WA-Budapest2023)

## Abstract

Major international sport events are often accompanied by facility development that is expected to generate benefits beyond the event period. This study examines residents’ perceptions of facility development related to the 2023 World Athletics Championships in Budapest, focusing on the National Athletics Centre. Using a questionnaire survey of 1,001 Budapest residents aged 18–89, representative of this population by gender and age, the study analyses perceived positive and negative impacts of the development and examines differences according to sociodemographic characteristics and forms of sport involvement. The analysis identified two dimensions of perceived impacts. Positive impacts referred to urban infrastructure, sport and leisure functions, and future sport participation opportunities, whereas negative impacts referred to expected underuse, preparation costs, and the perceived necessity of the investment. Sociodemographic characteristics were not associated with significant differences in perceptions. In contrast, following the event, being an athletics fan, and sport participation were associated with more favourable evaluations of positive impacts. The findings suggest that residents’ connection to sport and to the event-related discipline may be relevant for understanding how local communities evaluate event-related sport facility development and how benefits related to future use should be communicated.

## 1 Introduction

International sport events have long been associated with urban and infrastructural development, including the renewal of urban areas, the construction or renovation of sport facilities, the improvement of public spaces, and the expansion of leisure infrastructure (1, 2). The scale of such events varies considerably, with the largest commonly classified as mega-events (3). These event-related urban and infrastructural developments are commonly discussed as potential long-term outcomes of sport event hosting (4). Such developments are often used to justify event hosting, although previous research has questioned whether promised benefits are realised after the event, particularly in relation to public expenditure, cost escalation, and post-event use (5–8).

Sport facility planning and development are closely connected to public policy, financing, ownership, utilisation, and opportunities for sport participation. In local sport facility policy, these issues include the role of governance arrangements and power relations in shaping facility development (9), local sport policymaking and management processes (10), the relationship between facility provision and sport participation (11), and the utilisation of publicly supported facilities by clubs, athletes, recreational users, and local communities (12).

Over the past decade, Budapest has hosted several major international sport events, including the 2017 and 2022 World Aquatics Championships, UEFA Euro 2020 matches, the 2023 UEFA Europa League final, the 2023 World Athletics Championships, and the 2026 UEFA Champions League final (13–18). During the same period, Budapest was characterised by visible sport facility development, while several major venues—including the Duna Arena, the Puskás Aréna, and the National Athletics Centre—were used for international events (13–18). These developments took place in a national policy environment in which sport has been positioned as a strategic state sector, supported by public investment and sport facility and infrastructure development (19–21). Hungarian sport facility development between 2010 and 2016 has also been described as a period of expansion, and research on Budapest has linked sport facility development to political and institutional processes in the city (22, 23).

The National Athletics Centre was built in the southern part of Budapest, on the eastern bank of the Danube, as the main venue for WA-Budapest2023 (17, 24). Project and architectural descriptions refer to the athletics stadium site as a brownfield or rust-belt area connected to the former VITUKI industrial site and the wider Southern City Gate development area (25, 26). After the event, the athletics stadium was planned to include sport, recreational, and publicly accessible functions (25, 26). The temporary upper stands were removed after the Championships, and project descriptions indicate that its permanent post-event configuration was completed in 2024, including a public leisure ring and recreational functions around the venue (25, 26). The National Athletics Centre subsequently hosted the Gyulai István Memorial—Hungarian Athletics Grand Prix and is scheduled to host the World Athletics Ultimate Championship in 2026 (27, 28).

The hosting of major international sport events in Budapest has also been accompanied by public and political debate. The Nolimpia campaign related to Budapest's 2024 Olympic bid showed that sport event ambitions may become contested public issues, particularly in relation to public expenditure, urban priorities, and expected local benefits (29). The planning and development of the athletics stadium can therefore be situated within broader debates about public investment in sport facilities, municipal development priorities, and planned post-event use (21, 23, 25, 26).

Limited empirical evidence is available on how residents evaluate the planning, development, and expected use of event-related sport facilities in Hungary. Existing Hungarian studies have examined residents’ perceptions in relation to specific sport events, including the Győr European Youth Olympic Festival and UEFA Euro 2020 matches hosted in Budapest (30–32). These studies provide important national precedents for analysing residents’ views, but they did not focus specifically on the planning and development of a newly built sport facility.

Using survey data from adult residents of Budapest, the present study examines perceptions of the planning and development of the National Athletics Centre, focusing on perceived positive and negative impacts and on whether these perceptions differ according to sociodemographic characteristics and three forms of sport involvement: following WA-Budapest2023, being an athletics fan, and sport participation.

## 2 Literature review

### 2.1 Theoretical framing: social exchange theory and forms of sport involvement

Residents’ perceptions of event-related facility development can be interpreted through the logic of perceived benefits and costs. Social Exchange Theory has been widely used in tourism and sport event research to explain why residents evaluate events and their impacts positively or negatively (33–35). According to this perspective, residents are more likely to evaluate an event or a related development favourably when they perceive that its benefits outweigh its costs. Perceptions may become less favourable when the development is associated with excessive costs, limited benefits, or burdens that are not compensated by meaningful local gains (36).

This approach may be relevant to sport facility development as well. A new venue could be perceived as beneficial when residents associate it with urban renewal, improved recreational spaces, and future opportunities for sport participation (1, 2, 4, 8). The same development may be perceived negatively if it is considered too expensive or unlikely to be used after the event (5, 8, 36–38). From this perspective, perceived future use may be important in residents’ evaluations of a publicly supported sport facility development.

Residents’ evaluations may also depend on their personal relationship with sport. Sport involvement refers to the extent to which sport is personally relevant, interesting, or important to individuals (39). Previous studies have linked stronger sport or event involvement to more favourable perceptions of sport events and their impacts (31, 40–42).

### 2.2 Residents’ perceptions, sociodemographic characteristics and forms of sport involvement

Research on residents’ perceptions has developed into an important field within sport event studies. Previous studies have examined perceptions across different types of events, including the Olympic Games, the FIFA World Cup, Formula 1, the Tour de France, and other international or recurring sport events (42–50). Reviews and studies on measurement development have emphasised the importance of adapting measurement to the event type, host city context, and locally relevant impacts (36, 51, 52).

The dimensions examined also vary according to the timing of data collection and research purpose (36, 51–54). Recurring themes include economic and tourism benefits, community development, city image, cultural exchange, environmental issues, public expenditure, traffic, crowding, and infrastructure (36, 51–53). This variation supports the use of context-specific measurement approaches, particularly when the focus shifts from the event itself to an event-related sport facility.

Previous research has also examined whether perceptions differ according to gender, age, and educational attainment. The findings are mixed. Some studies found differences by age, gender, or education, whereas others found no significant sociodemographic differences (43–46, 48–50, 55). This inconsistency suggests that sociodemographic characteristics are useful comparison variables, but their effects should not be assumed to be uniform across events and contexts.

Empirical research has examined different forms of sport and event involvement and has generally linked stronger involvement to more favourable impact perceptions or stronger event support, although the relationships may vary according to the form of involvement and the event context (31, 40–42).

### 2.3 Event-related sport facility development and post-event use

Sport facility development is one of the most visible outcomes of hosting major international sport events. New or renovated stadiums, arenas, training centres, and related service facilities may contribute to sport infrastructure, urban regeneration, public spaces, and leisure opportunities (1, 2, 4). From a broader territorial development perspective, sport events can also affect a range of territorial resources in host places, which makes their evaluation more complex than the measurement of event-time impacts alone (56).

Facility development is closely linked to the long-term outcomes of sport event hosting (5, 57–59). These outcomes may remain after the event and may be planned or unplanned, positive or negative, tangible or intangible (5, 57, 58). Infrastructure-related outcomes are among the most visible forms of such changes, but a venue's long-term value depends on whether post-event use is integrated into bidding, planning, design, and operation (5, 37, 57, 58).

Costs, maintenance, and utilisation are central issues in this debate. Public subsidies for stadiums and event-related venues have been widely discussed in sport economics and public policy, particularly when construction costs, operating costs, or opportunity costs are high (21, 38). Research on mega-event costs has also shown that large sport projects can be exposed to cost overruns and financial risk (60). If long-term use is limited, venues may become underused or financially burdensome, a problem often discussed through the concept of “white elephants” (37).

Sustainable sport facility development requires attention to environmental, social, and economic dimensions (61). Event-related sport facilities may contribute to urban regeneration when they are integrated into surrounding urban functions and public spaces, but such effects are not automatic (1, 2, 4).

Previous empirical studies show that facility and infrastructure development are important components of residents’ perceptions. Studies on the FIFA World Cup, Formula 1, and other events have shown that residents may recognise infrastructure-related benefits while also expressing concerns about construction costs, economic burdens, and post-event stadium use (43–50). These findings indicate that facility development is often evaluated through the perceived balance between local benefits and costs.

Beyond event-related studies, a limited number of studies have focused specifically on residents’ perceptions of sport facilities. Research on neighbourhood sport centres has reported generally favourable evaluations concerning health, physical activity, social cohesion, and urban development and infrastructure (62, 63), with perceptions varying according to facility use and place of residence (63). Studies of larger municipal and publicly subsidised facilities have likewise reported perceived benefits related to accessibility, sport participation, community image, and community development, while also considering concerns about costs, traffic-related problems, and whether such benefits extend beyond users to the wider community (64, 65). These studies further point to the relevance of residents’ relationship with the facility and the specific local context.

### 2.4 Analytical framework and research questions

The analytical framework of this study is based on Social Exchange Theory and forms of sport involvement. Social Exchange Theory provides a basis for interpreting residents’ evaluations of event-related facility development through perceived benefits and costs (33–35). In this study, perceived benefits refer to urban infrastructure development, sport and leisure functions, and future sport participation opportunities, while the negative dimension concerns preparation costs, expected underuse, and doubts about the necessity of the investment.

Forms of sport involvement are included because previous studies indicate that residents’ involvement in sport, the event, or the related sport may be associated with more favourable evaluations of sport event impacts (31, 40–42). In the Hungarian context, studies on Euro 2020 identified differences in residents’ perceptions according to some sociodemographic characteristics and forms of sport involvement. Although perceptions of both positive and negative impacts decreased after the event, several of these differences remained (31, 32). These findings support the relevance of examining whether similar patterns can be observed in relation to facility development connected to WA-Budapest2023.

This approach is relevant from a facility development policy perspective, in which governance, sport participation, and facility utilisation are central considerations (9–12). Previous studies indicate that residents’ evaluations may vary according to their involvement in sport or the event and, in facility-based research, according to their relationship with the facility (40–42, 63). Identifying such differences may help decision-makers understand how different resident groups evaluate the facility and inform communication about its post-event functions. In the case of facilities developed for elite sport events, an important policy question is how their post-event functions can support recreational sport and wider local use (5, 12, 37, 64, 65).

Based on this framework, the study addresses the following research questions:

**RQ1:** How do Budapest residents evaluate the perceived positive and negative impacts of facility development related to the National Athletics Centre?
**RQ2:** Do residents’ perceptions of the positive and negative impacts differ according to sociodemographic characteristics?
**RQ3:** Do residents’ perceptions of the positive and negative impacts differ according to forms of sport involvement, including following WA-Budapest2023, being an athletics fan, and sport participation?
**RQ4:** Which sociodemographic characteristics and forms of sport involvement predict perceived positive and negative impacts when examined simultaneously?

## 3 Materials and methods

### 3.1 Research design and data collection

The study is based on a cross-sectional questionnaire survey conducted among Budapest residents aged 18–89. Data collection took place three months after WA-Budapest2023. This timing made it possible to examine residents’ perceptions after the event, when the Championships had already been experienced by the host city and public discussion about the facility development and its post-event use remained close in time to data collection. The survey focused on residents’ perceptions of the newly built National Athletics Centre, which was developed in connection with WA-Budapest2023.

### 3.2 Sample

The final sample consisted of 1,001 Budapest residents aged 18–89. The sample was representative of the Budapest population aged 18–89 by gender and age. Men represented 44.4% of the sample, compared with 45.1% in this population, while women represented 55.6% of the sample, compared with 54.9%.

Educational attainment showed a less balanced distribution. Respondents with primary education accounted for 17.8% of the sample, which was close to the population proportion of 17.0%. However, respondents with secondary education were overrepresented in the sample, while respondents with higher education were underrepresented compared with the corresponding Budapest population. The detailed comparison between the sample and the Budapest population aged 18–89, based on a custom tabulation supplied by the Hungarian Central Statistical Office from the 2011 Population Census, is presented in Table 1 (66).

**Table 1:** Sample characteristics and Budapest population aged 18–89 (*N* = 1,001).

Variables	Sample, *n* (%)	Adult population of Budapest aged 18–89, *N* = 1,464,739 (%)
Gender		
Male	444 (44.4%)	659,932 (45.1%)
Female	557 (55.6%)	804,807 (54.9%)
Age group (years)		
18–29	196 (19.6%)	285,050 (19.5%)
30–39	210 (21.0%)	313,540 (21.4%)
40–49	143 (14.3%)	213,367 (14.6%)
50–59	154 (15.4%)	225,379 (15.4%)
60–69	148 (14.8%)	216,828 (14.8%)
70–89	150 (15.0%)	210,575 (14.3%)
Educational attainment		
Primary education	178 (17.8%)	249,487 (17.0%)
Secondary education	670 (66.9%)	749,341 (51.2%)
Higher education	153 (15.3%)	465,911 (31.8%)

Data refer to the population of Budapest aged 18–89 and were supplied by the Hungarian Central Statistical Office in a custom tabulation based on the 2011 Population Census (66).

### 3.3 Measures

Residents’ perceptions of facility development were measured with six statements related to WA-Budapest2023 and the National Athletics Centre. The item set was informed by previous residents’ perception instruments measuring positive and negative event impacts (51, 52). The items concerning preparation costs and future underuse were adapted from earlier measures (51), while the items concerning urban infrastructure, post-event sport and leisure functions, future sport participation opportunities, and the perceived necessity of the investment were formulated for the specific context of WA-Budapest2023 and the National Athletics Centre. Respondents indicated their agreement with each statement on a five-point Likert scale ranging from 1 (“strongly disagree”) to 5 (“strongly agree”).

The three positive impact items were as follows: 1) “The event-related facility development contributed to the general development of urban infrastructure in Budapest”; 2) “The new stadium will function as a sport and leisure centre and recreational park”; and 3) “The facility development will create future sport participation opportunities for local residents”. The three negative impact items were as follows: 1) “The National Athletics Centre is expected to be underused in the future”; 2) “The preparation costs related to the facility development were excessive”; and 3) “It would have been better if the facility development had not been implemented”.

For the subsequent analyses, the three positive items and the three negative items were averaged separately to create two composite scores: perceived positive impacts and perceived negative impacts. Higher scores indicated stronger perceived positive or negative impacts, respectively.

Sociodemographic variables included gender, age, and educational attainment. Forms of sport involvement were measured using three variables: following WA-Budapest2023, being an athletics fan, and sport participation.

Following the event was measured by asking whether respondents had followed WA-Budapest2023 in person or through media channels, including television, radio, newspapers, online platforms, or social media. Responses were recoded into a dichotomous variable distinguishing those who followed the event from those who did not. Being an athletics fan was measured with a dichotomous question offering two response options: “yes” and “no”.

Sport participation was measured by asking respondents how often they had participated in sport during the twelve months preceding data collection. The response options were: five times per week or more, three to four times per week, one to two times per week, and never. For the analysis, this variable was recoded into a dichotomous variable. Respondents who reported sport participation one to two times per week or more were coded as “yes”, while those who selected “never” were coded as “no”. The distribution of the three sport involvement variables—following WA-Budapest2023, being an athletics fan, and sport participation—is presented in Table 2.

**Table 2:** Forms of sport involvement (*N* = 1,001).

Variable/response	*N* (%)
Following the event	
Yes	560 (55.9%)
No	441 (44.1%)
Fan of athletics	
Yes	520 (51.9%)
No	481 (48.1%)
Sport participation	
Yes	321 (32.1%)
No	680 (67.9%)

### 3.4 Data analysis

Statistical analyses were performed using IBM SPSS Statistics 27, while confirmatory factor analysis was conducted using IBM SPSS Amos 26. First, descriptive statistics were calculated to present the sample characteristics, forms of sport involvement, item-level responses, and perceived impact scores.

Second, confirmatory factor analysis (CFA) was conducted to assess the hypothesised two-factor structure of the six facility development impact items. Three items were specified to load on the perceived positive impacts factor and three items on the perceived negative impacts factor, and the two latent factors were allowed to correlate. The model was estimated using maximum likelihood estimation. Model fit was evaluated using the chi-square statistic, the chi-square to degrees of freedom ratio (*χ*^2^/df), the comparative fit index (CFI), the Tucker–Lewis index (TLI), the root mean square error of approximation (RMSEA), and the standardised root mean square residual (SRMR). Values below 3 for *χ*^2^/df, values of .90 or higher for CFI and TLI, and values of .08 or lower for RMSEA and SRMR were considered indicative of acceptable model fit (67). The hypothesised two-factor CFA model showed an acceptable fit to the data: *χ*^2^(8) = 20.69, *p* = .008, *χ*^2^/df = 2.59, CFI = .959, TLI = .923, RMSEA = .040, and SRMR = .049. Standardised factor loadings ranged from .76 to .85 for perceived positive impacts and from .71 to .83 for perceived negative impacts.

Cronbach's alpha and composite reliability (CR) were calculated to assess internal consistency and construct reliability, while average variance extracted (AVE) was used to assess convergent validity. Values of .70 or higher for Cronbach's alpha and CR and .50 or higher for AVE were considered acceptable. The positive impact factor showed acceptable internal consistency, construct reliability, and convergent validity (*α* = .84; CR = .84; AVE = .63). The negative impact factor also showed acceptable internal consistency, construct reliability, and convergent validity (*α* = .77; CR = .79; AVE = .56). Discriminant validity was assessed using the Fornell–Larcker criterion. The squared correlation between the two latent factors was lower than the AVE values of both factors, supporting discriminant validity (68).

Third, group differences in perceived positive and negative impacts were examined according to sociodemographic characteristics and forms of sport involvement. Independent-samples *t*-tests were used for binary variables, including gender, following the event, being an athletics fan, and sport participation. One-way analysis of variance was used for variables with more than two categories, including age group and respondents’ highest level of educational attainment. Homogeneity of variances was assessed using Levene's test. When this assumption was violated, the unequal-variances form of the independent-samples *t*-test was reported for two-group comparisons, while Welch's analysis of variance was used for comparisons involving more than two groups.

Effect sizes were reported as Cohen's *d* for independent-samples *t*-tests and eta squared (*η*^2^) for one-way analyses of variance. Absolute values of 0.20, 0.50, and 0.80 for Cohen's *d* and values of 0.01, 0.06, and 0.14 for *η*^2^ were interpreted as small, medium, and large effects, respectively (69).

Finally, separate linear regression models were estimated to examine the predictors of perceived positive and negative impacts. Gender was coded 0 = male and 1 = female. Following the event, being an athletics fan, and sport participation were coded 0 = no and 1 = yes. Age measured in years was entered as a continuous predictor, while respondents’ highest level of educational attainment was entered as an ordinal predictor in ascending order.

Regression diagnostics included tolerance and variance inflation factor (VIF) values, assessments of linearity and residual normality, the Breusch–Pagan and White tests for heteroscedasticity (70, 71), and Cook's distance. Tolerance values ranged from .684 to .941 and VIF values from 1.064 to 1.462, indicating no multicollinearity. The linearity assumption was considered acceptable, the residuals were approximately normally distributed, and Cook's distance values indicated no unduly influential observations. Both the Breusch–Pagan and White tests were statistically significant for the positive and negative impact models (all *p* < .001), indicating heteroscedasticity. Consequently, HC3 heteroscedasticity-consistent standard errors were used to calculate the standard errors and *p*-values of the regression coefficients (72, 73). All independent-samples *t*-tests were two-sided, and statistical significance was set at *p* < .05 for the group comparisons and regression analyses.

## 4 Results

### 4.1 Dimensions and descriptive statistics of perceived facility development impacts

The descriptive statistics for the perceived facility development impact items are presented in Table 3. The items were grouped into two dimensions: perceived positive impacts and perceived negative impacts.

**Table 3:** Descriptive statistics and measurement properties of perceived facility development impacts (*N* = 1,001).

Dimension/item	M (SD)	*λ*
Positive impacts (*α* = .84; CR = .84; AVE = .63)		
Development of general urban infrastructure	3.34 (1.15)	.85
Stadium, sport and leisure centre, and recreational park	3.53 (1.15)	.79
Future sport participation opportunities for local residents	3.20 (1.12)	.76
Negative impacts (*α* = .77; CR = .79; AVE = .56)		
Expected underuse of the facility	3.23 (1.18)	.83
Excessive preparation costs	3.43 (1.00)	.72
It would have been better if the facility development had not been implemented	2.97 (1.16)	.71

M, mean; SD, standard deviation; *λ*, standardised factor loading; *α*, Cronbach's alpha; CR, composite reliability; AVE, average variance extracted. The hypothesised two-factor CFA model showed an acceptable fit to the data: *χ*^2^(8) = 20.69, *p* = .008, *χ*^2^/df = 2.59, CFI = .959, TLI = .923, RMSEA = .040, and SRMR = .049.

The positive impact dimension comprised three items: development of general urban infrastructure; development of the stadium, sport and leisure centre, and recreational park; and future sport participation opportunities for local residents. The highest mean score was recorded for the item concerning the stadium, sport and leisure centre, and recreational park (M = 3.53, SD = 1.15), followed by development of general urban infrastructure (M = 3.34, SD = 1.15) and future sport participation opportunities for local residents (M = 3.20, SD = 1.12).

The negative impact dimension also comprised three items: expected underuse of the facility, excessive preparation costs, and the view that it would have been better if the facility development had not been implemented. The highest mean score was recorded for excessive preparation costs (M = 3.43, SD = 1.00), followed by expected underuse of the facility (M = 3.23, SD = 1.18) and the view that it would have been better if the facility development had not been implemented (M = 2.97, SD = 1.16).

### 4.2 Differences by sociodemographic characteristics

Differences in perceived positive and negative impacts by sociodemographic characteristics are presented in Table 4.

**Table 4:** Differences in perceived facility development impacts by sociodemographic characteristics (*N* = 1,001).

Variable/category	Perceived positive impacts M (SD)	Perceived negative impacts M (SD)
Gender		
Male	3.42 (0.92)	3.15 (0.81)
Female	3.31 (0.94)	3.26 (0.85)
Test statistic	*t* = 1.95; *d* = 0.12	*t* = −1.95; *d* = −0.12
Age group (years)		
18–29	3.37 (0.84)	3.11 (0.84)
30–39	3.42 (0.96)	3.18 (0.72)
40–49	3.45 (0.90)	3.28 (0.83)
50–59	3.34 (0.90)	3.20 (0.79)
60–69	3.36 (0.90)	3.27 (0.89)
70–89	3.19 (0.96)	3.26 (0.97)
Test statistic	*F* = 1.46; *η*^2^ = .007	*F* = 1.14; *η*^2^ = .006
Educational attainment		
Primary education	3.28 (0.84)	3.21 (0.91)
Secondary education	3.37 (0.88)	3.20 (0.80)
Higher education	3.41 (0.90)	3.24 (0.90)
Test statistic	*F* = 0.87; *η*^2^ = .002	*F* = 0.10; *η*^2^ < .001

M, mean; SD, standard deviation; *d*, Cohen's *d*; *η*^2^, eta squared. For Cohen's *d*, absolute values of 0.20, 0.50, and 0.80 indicate small, medium, and large effects, respectively; for *η*^2^, values of .01, .06, and .14 indicate small, medium, and large effects, respectively.

No statistically significant differences were found by gender. Men reported slightly higher perceived positive impacts (M *=* 3.42, SD *=* 0.92) than women (M = 3.31, SD = 0.94), while women reported slightly higher perceived negative impacts (M = 3.26, SD = 0.85) than men (M *=* 3.15, SD *=* 0.81). Neither difference was statistically significant, and both effect sizes were negligible: positive impacts (*t* = 1.95, *p* = .051, Cohen's *d* = 0.12) and negative impacts (*t* = −1.95, *p* = .052, Cohen's *d* = −0.12).

No significant differences were found by age group, and the effect sizes were negligible. Mean scores for perceived positive impacts ranged from 3.19 to 3.45 across age groups (*F* = 1.46, *p* = .201, *η*^2^ = .007), while mean scores for perceived negative impacts ranged from 3.11 to 3.28 (*F* = 1.14, *p* = .340, *η*^2^ = .006).

Educational attainment was also not associated with statistically significant differences in perceived impacts, with negligible effect sizes. Mean scores for perceived positive impacts ranged from 3.28 to 3.41 across educational groups (*F* = 0.87, *p* = .420, *η*^2^ = .002), while mean scores for perceived negative impacts ranged from 3.20 to 3.24 (*F* = 0.10, *p* = .904, *η*^2^ < .001).

### 4.3 Differences by forms of sport involvement

Differences in perceived positive and negative impacts by following WA-Budapest2023, being an athletics fan, and sport participation are reported in Table 5.

**Table 5:** Differences in perceived facility development impacts by sport involvement (*N* = 1,001).

Variable/category	Perceived positive impacts M (SD)	Perceived negative impacts M (SD)
Following the event		
Yes	3.61 (0.78)	3.11 (0.79)
No	3.05 (1.01)	3.34 (0.88)
Test statistic	*t* = 9.63***; *d* = 0.63	*t* = −4.48***; *d* = −0.29
Fan of athletics		
Yes	3.72 (0.70)	3.15 (0.75)
No	2.97 (0.99)	3.28 (0.92)
Test statistic	*t* = 13.63***; *d* = 0.87	*t* = −2.38*; *d* = −0.15
Sport participation		
Yes	3.69 (0.83)	3.15 (0.84)
No	3.20 (0.93)	3.24 (0.83)
Test statistic	*t* = 8.27***; *d* = 0.54	*t* = −1.65; *d* = −0.11

M, mean; SD, standard deviation; *d*, Cohen's *d*. Absolute values of 0.20, 0.50, and 0.80 indicate small, medium, and large effects, respectively.

*p < .05, **p < .01, ***p < .001.

Regarding forms of sport involvement, 55.9% of respondents reported following WA-Budapest2023, 51.9% reported being an athletics fan, and 32.1% reported sport participation. Group comparisons showed significant differences in perceived positive and negative impacts according to several forms of sport involvement.

Following WA-Budapest2023 was associated with significant differences in both perceived positive and negative impacts. Respondents who followed the event reported higher perceived positive impacts (M = 3.61, SD = 0.78) than those who did not follow the event (M = 3.05, SD = 1.01; *t* = 9.63, *p* < .001, Cohen's *d* = 0.63). They also reported lower perceived negative impacts (M = 3.11, SD = 0.79) than respondents who did not follow the event (M = 3.34, SD = 0.88; *t* = −4.48, *p* < .001, Cohen's *d* = −0.29).

Being an athletics fan was also associated with significant differences in both dimensions. Respondents who were athletics fans reported higher perceived positive impacts (M = 3.72, SD = 0.70) than those who were not athletics fans (M = 2.97, SD = 0.99; *t* = 13.63, *p* < .001, Cohen's *d* = 0.87). They also reported lower perceived negative impacts (M = 3.15, SD = 0.75) than respondents who were not athletics fans (M = 3.28, SD = 0.92; *t* = −2.38, *p* = .018, Cohen's *d* = −0.15).

Respondents who reported sport participation had significantly higher scores for perceived positive impacts (M = 3.69, SD = 0.83) than those who did not report sport participation (M = 3.20, SD = 0.93; *t* = 8.27, *p* < .001, Cohen's *d* = 0.54). No statistically significant difference was found for perceived negative impacts by sport participation (*t* = −1.65, *p* = .100, Cohen's *d* = −0.11).

### 4.4 Regression models predicting perceived positive and negative impacts

The regression models predicting perceived positive and negative impacts of facility development are presented in Table 6. The model predicting perceived positive impacts explained 20.0% of the variance (R^2^ = .200). Following the event (*b* = 0.304, *β* = .151, SE = 0.065, *p* < .001), being an athletics fan (*b* = 0.572, *β* = .286, SE = 0.064, *p* < .001), and sport participation (*b* = 0.364, *β* = .170, SE = 0.069, *p* < .001) were significant positive predictors. Gender, age, and educational attainment were not significant predictors of perceived positive impacts.

**Table 6:** Linear regression models predicting perceived positive and negative impacts of facility development (*N* = 1,001).

Variables	Positive impacts				Negative impacts			
	*b*	*β*	SE	*p*	*b*	*β*	SE	*p*
Gender	0.047	.023	0.058	.419	0.054	.027	0.062	.387
Age	0.002	.032	0.002	.326	0.003	.044	0.002	.231
Educational attainment	0.012	.007	0.053	.820	0.039	.022	0.060	.519
Following the event	0.304	.151	0.065	<.001	−0.267	−.133	0.066	<.001
Fan of athletics	0.572	.286	0.064	<.001	0.024	.012	0.068	.725
Sport participation	0.364	.170	0.069	<.001	−0.067	−.031	0.076	.379
R^2^	.200				.024			

*b*, unstandardised regression coefficient; *β*, standardised regression coefficient; SE, HC3 heteroscedasticity-consistent standard error. Reported *p*-values are based on HC3 robust standard errors.

In the model predicting perceived negative impacts, the included variables explained 2.4% of the variance (R^2^ = .024). Following the event was the only significant predictor (*b* = −0.267, *β* = −.133, SE = 0.066, *p* < .001). Gender, age, educational attainment, being an athletics fan, and sport participation were not significant predictors of perceived negative impacts.

## 5 Discussion and conclusion

This study examined how Budapest residents evaluated the planning and development of the newly built National Athletics Centre in connection with hosting a major international sport event. The findings show that residents’ opinions were constructed around both favourable and critical evaluations. This pattern indicates that the facility was not assessed in a single direction: potential local benefits were considered alongside concerns about costs, future use, and the necessity of the investment.

The interpretation of results is consistent with Social Exchange Theory, which explains residents’ evaluations through the perceived balance between benefits and costs (33–35). In the present study, positive evaluations were connected to urban development, recreational and sport-related functions, and future use, while critical evaluations were linked to financial burden, possible underuse, and doubts about the necessity of the development. The separation of positive and negative dimensions is also in line with previous research on residents’ perceptions, where benefits and costs have often been analysed as distinct dimensions of evaluation (36, 51, 52, 65, 74).

Post-event use is central to understanding residents’ opinions on the National Athletics Centre. Research on local sport facility planning has emphasised that facility development is shaped by policy priorities, governance arrangements, and power relations (9). Other studies have connected facility provision to sport participation and local sport policy (10, 11), while utilisation has been identified as a policy and management issue for publicly supported sport facilities (12). The findings can be read alongside this literature, as residents’ evaluations were partly related to concerns about possible underuse, recreational functions, urban infrastructure, and future sport participation opportunities for local residents.

Basic sociodemographic characteristics did not clearly differentiate residents’ evaluations of the National Athletics Centre. This result differs from studies in which demographic variables explained variation in residents’ perceptions, but it is consistent with research showing that such patterns are often inconsistent and may depend on the event, host city, measured dimensions, and sample composition (43–46, 48–50, 55). It also differs from earlier Hungarian research on Euro 2020, where differences in residents’ perceptions remained visible across some sociodemographic and sport involvement variables after the event (31, 32). The absence of significant differences by age and educational attainment is consistent with the findings of Testa et al. (63).

Forms of sport involvement showed a clearer pattern. This finding is consistent with sport involvement theory, which links involvement to the personal relevance, interest, and importance attached to sport (39). It is also in line with previous studies linking stronger sport or event involvement to more favourable evaluations (40–42). A comparable pattern was found in a study of a neighbourhood sport centre, where users reported more favourable evaluations across the measured dimensions than non-users (63). Being an athletics fan is especially relevant in this case, as the National Athletics Centre was developed for athletics and has subsequently hosted and is scheduled to host further international athletics events (27, 28). At the same time, sport involvement should not be interpreted as the only factor behind residents’ opinions. Rather, it may help explain why some groups recognise the sport and recreational functions of the facility more positively.

The negative dimension followed a different pattern. Concerns about costs, underuse, and the necessity of the investment may be shaped by factors beyond the sociodemographic and sport involvement variables included in this study. These factors may include views on public expenditure, trust in facility planning, knowledge about post-event use, and the availability of clear resident information about post-event and recreational functions. Such concerns correspond with previous research in which costs, underuse, and facility utilisation appeared as recurring issues in the evaluation of event-related sport facility development and publicly supported sport facilities (12, 21, 37, 38, 43, 47–49). Concerns about costs in the present study are also consistent with research on municipal and publicly subsidised sport facilities, which considered financial burdens and traffic-related problems among the concerns associated with facility development (64, 65).

The study has practical implications for facility planning and development. Facilities developed for elite sport events may gain broader local value when residents are clearly informed about their post-event use and recreational functions, particularly when such facilities are financed or supported through public resources. The findings also show that sport-involved residents evaluated positive impacts more favourably, suggesting that communication about the facility's wider urban and recreational functions may be particularly important for residents with weaker connections to sport.

The study contributes to the limited empirical evidence on residents’ evaluations of event-related sport facility planning and development. Its specific contribution lies in the focus on a newly built athletics facility in Budapest and in the use of survey data from adult residents of the city. By examining positive and negative perceptions together with sociodemographic characteristics and forms of sport involvement, the study provides evidence on how local residents evaluated the National Athletics Centre shortly after its first major international event.

## 6 Limitations and future research

Although this research analysed local residents’ perceptions using a sample whose gender and age distribution closely reflected the population data used for comparison, it also has several limitations.

First, the data were collected at one point in time, three months after the event; therefore, the analysis cannot show how residents’ perceptions changed in comparison with the pre-event period. Future studies could apply longitudinal designs to examine the stability or change of perceptions over a longer period.

Second, the analysis focused on one newly built athletics facility in Budapest, the capital of Hungary, and therefore reflects a specific national and urban context. The findings should not be generalised without caution to other sport facilities, host cities, or national contexts. Comparative research across different types of facilities and host settings would help to clarify which findings are context-specific and which may be more general.

Third, forms of sport involvement were measured using three dichotomous variables. Future research could examine sport involvement in greater detail by using multidimensional scales covering event following, being an athletics fan, sport participation, media consumption, and sport-specific interest. This would provide a more detailed understanding of how residents’ relationships with sport shape their perceptions of the planning and development of sport infrastructure connected to the hosting of international sport events.

Fourth, the study did not include socioeconomic and territorial variables such as household income and length of residence (65), residential location (63), or proximity to the National Athletics Centre. Including these variables in future research may provide a more detailed understanding of potential differences in residents’ perceptions.

Despite these limitations, the study adds useful empirical evidence to the limited Hungarian and international literature on residents’ perceptions of event-related facility development. To the authors’ knowledge, it is among the first studies to focus specifically on residents’ evaluations of the planning and development of a sport facility connected to a major international sport event in Budapest and to use a sample representative of the Budapest population aged 18–89 by gender and age. By examining perceived positive and negative impacts and differences by sociodemographic characteristics and forms of sport involvement, the study offers evidence that may be relevant for future policies and approaches to the planning and development of sport facilities connected to international sport events. The findings underline the importance of providing residents with clear information about post-event use, access, costs, and recreational sport opportunities. Facilities developed for elite sport events may gain broader social relevance when their future functions are also connected to everyday sport participation and community use.

## Data Availability

The original contributions presented in the study are included in the article, further inquiries can be directed to the corresponding author.
